# Stigmatizing attitudes of doctors, practicing psychiatry in Slovenia; Eustigma study results

**DOI:** 10.1192/j.eurpsy.2024.689

**Published:** 2024-08-27

**Authors:** P. Rus Prelog, A. Mirkovič, D. Őri

**Affiliations:** ^1^ Centre for Clinical Psychiatry, University Psychiatric Clinic LJubljana; ^2^Child Psychiatry Unit, University Children’s Hospital, Child Psychiatry Unit, University Medical Centre Ljubljana, Ljubljana, Slovenia; ^3^Institute of Behavioural Sciences, Semmelweis University; ^4^Department of Mental Health, Heim Pal National Pediatric Institute, Budapest, Hungary

## Abstract

**Introduction:**

The perception that individuals afflicted with mental disorders may exhibit potential harm or unpredictability is common in the general population and, as studies have shown, mental health-related stigma is not confined to the broader public but is progressively emerging as a concern within professional circles as well, adding additional burden to patients in psychiatric settings who already encounter an array of impediments stemming from societal prejudice.

**Objectives:**

In this cross-sectional study, we aimed to investigate the attitudes of adult and child psychiatrists towards people with mental health problems in Slovenia.

**Methods:**

The stigmatizing attitudes were measured by an internet-based, anonymous survey using the Opening Minds Stigma Scale for Health Care Providers (total score and three subscales are the following: attitude, disclosure and help-seeking, social distance).

**Results:**

Altogether, n=90 practitioners (n=18 males, n=72 females) completed the survey. The bifactor ESEM model showed the best model fit (RMSEA=0.060, CFI=0.970, TLI=0.939); however, exploratory factor analysis results indicated the weakness of items 1 and 11. Those participants who have a possibility to attend case discussion groups are more willing to disclose their own mental health issues or seek help (8 (7-9) vs 9 (8-11.5)); however, they prefer more social distance from their patients (9(7.5-10) vs 7(6-9)). Gender differences were found as well, women seem to keep more social distance (p=0.031). Interestingly, those practitioners who reported spending 75% of their working hours with patients kept less social distance compared to those who engage in other activities (p=0.028).

**Conclusions:**

This study is the first to describe the stigmatizing attitude of psychiatric practitioners in Slovenia from their perspective, and it provides directions for anti-stigma interventions.

**Disclosure of Interest:**

None Declared

